# *Pleurotus giganteus* (Berk.) Karunarathna & K.D. Hyde: Nutritional value and *in vitro* neurite outgrowth activity in rat pheochromocytoma cells

**DOI:** 10.1186/1472-6882-12-102

**Published:** 2012-07-19

**Authors:** Chia-Wei Phan, Wei-Lun Wong, Pamela David, Murali Naidu, Vikineswary Sabaratnam

**Affiliations:** 1 Mushroom Research Centre, Institute of Biological Sciences, Faculty of Science, University of Malaya, 50603, Kuala Lumpur, Malaysia; 2 Institute of Biological Sciences, Faculty of Science, University of Malaya, 50603, Kuala Lumpur, Malaysia; 3 Department of Anatomy, Faculty of Medicine, University of Malaya, 50603, Kuala Lumpur, Malaysia

**Keywords:** *Pleurotus giganteus*, Medicinal mushroom, Edible mushroom, Neurite outgrowth, Neurodegenerative disease, MEK/ERK signalling pathway, PI3K/Akt signalling pathway

## Abstract

**Background:**

Drugs dedicated to alleviate neurodegenerative diseases like Parkinson’s and Alzheimer’s have always been associated with debilitating side effects. Medicinal mushrooms which harness neuropharmacological compounds offer a potential possibility for protection against such diseases. *Pleurotus giganteus* (formerly known as *Panus giganteus*) has been consumed by the indigenous people in Peninsular Malaysia for many years. Domestication of this wild mushroom is gaining popularity but to our knowledge, medicinal properties reported for this culinary mushroom are minimal.

**Methods:**

The fruiting bodies *P. giganteus* were analysed for its nutritional values. Cytotoxicity of the mushroom’s aqueous and ethanolic extracts towards PC12, a rat pheochromocytoma cell line was assessed by using 3-[4,5-dimethythiazol-2-yl]-2,5-diphenyltetrazolium bromide (MTT) assay. Neurite outgrowth stimulation assay was carried out with nerve growth factor (NGF) as control. To elucidate signaling mechanisms involved by mushroom extract-induced neurite outgrowth, treatment of specific inhibitor for MEK/ERK and PI3K signalling pathway was carried out.

**Results:**

The fruiting bodies of *P. giganteus* were found to have high carbohydrate, dietary fibre, potassium, phenolic compounds and triterpenoids. Both aqueous and ethanolic extracts induced neurite outgrowth of PC12 cells in a dose- and time-dependant manner with no detectable cytotoxic effect. At day 3, 25 μg/ml of aqueous extract and 15 μg/ml of ethanolic extract showed the highest percentage of neurite-bearing cells, i.e. 31.7 ± 1.1% and 33.3 ± 0.9%; respectively. Inhibition treatment results suggested that MEK/ERK and PI3K/Akt are responsible for neurite outgrowth of PC12 cells stimulated by *P. giganteus* extract. The high potassium content (1345.7 mg/100 g) may be responsible for promoting neurite extension, too.

**Conclusions:**

*P. giganteus* contains bioactive compounds that mimic NGF and are responsible for neurite stimulation. Hence, this mushroom may be developed as a nutraceutical for the mitigation of neurodegenerative diseases.

## Background

Neurodegenerative diseases are on the rise. The most common form of neurodegenerative disease is Alzheimer's disease, which causes thinking and memory to become seriously impaired due to neuronal loss in brain [1]. The second most common neurodegenerative syndrome, Parkinson's disease is characterised by the classic symptoms of tremors, rigidity and gait impairment [2]. Medications to alleviate these neurodegenerative diseases can only provide benefits for several years but are not effective as the diseases progress [3]. Some undesired side effects associated with these drugs include hallucinations, dyskinesia, nausea and constipation [2,3]. In this regard, complementary and alternative medicine which is now gaining momentum may be a promising way for prevention and protection against such neurodegenerative diseases [4].

Mushrooms are largely consumed not only in Asian countries but across Western countries. Mushrooms are of considerable interest because of their organoleptic merit, medicinal properties and economic significance. We have documented the effects of an edible mushroom *Hericium erinaceus* (Bull.:Fr.) Pers. (also known as monkey’s head, lion’s mane, and yamabushitake) on neurite outgrowth and peripheral nerve regeneration both *in vitro* and *in vivo*[5-7]. More recently, the aqueous extracts of *Lignosus rhinoceros* (Cooke) Ryvarden (tiger’s milk mushroom) was reported to induce neurite outgrowth with or without the trigger of nerve growth factor (NGF) [8]. The cocktail of bioactive compounds present in these mushroom extracts exhibits NGF-like properties and play important roles in the growth, differentiation and survival of neuronal cells [9-11].

Formerly known as *Panus giganteus* (Berk) Corner, *Pleurotus giganteus* (Berk.) Karunarathna & K.D. Hyde is a culinary mushroom that is gaining popularity for its organoleptic properties and commercial prospects. In fact, consumption of this used-to-be wild mushroom has long been a tradition in the indigenous villages in Peninsular Malaysia [12]. A variety of *P. giganteus* from China is now being cultivated in Malaysia and the common commercial name in Malay language for *P. giganteus* is “*Seri Pagi*” (morning glory). In China, *P. giganteus* is widely referred as “Zhudugu” (swine’s stomach) [13]. It is noteworthy to mention that the “*Panus-PleurotusLentinus”* complex has long existed and has resulted in the confusion of nomenclature and taxonomy of these three species. Briefly, *Panus giganteus* (Polyporaceae, Polyporales) is characterised by its unbranched skeletal hyphae that usually grow on buried woody substrates [14]. While Pegler [15] has merged *Panus* as a subgenus within *Lentinus*, Corner [14], has grouped the genus *Panus* to species with skeletal hyphae and separated those species with ligative hyphae in *Lentinus*. Hence, *Lentinus giganteus* is regarded as synonym for *Panus giganteus* and *Lentinus giganteus* should be used if recommendation of Pegler is ever adopted [13,16]. However, Karunarathna and colleagues have revisited this issue and concluded that *Panus*/*Lentinus giganteus* should be unified and positioned in *Pleurotus* as supported by molecular evidences [17].

Cell cultures derived from nervous system tissue have proven to be powerful tools for elucidating cellular mechanisms of nervous system function [18]. The effect of chemicals, drugs, natural products or even growth factors on neurite outgrowth can be quantified by enumerating the number of cells that bear neurites using *in vitro* cell line model [19]. Neurite refers collectively to “axons and dendrites extended by primary cells growing in culture, or processes extended by neuronal cell lines, which are neither definitive axons, nor dendrites” [19,20]. Pheochromocytoma (PC12) cells, originated from a rat adrenal medullary tumour (pheochromocytoma) have been widely employed as a model of neuronal differentiation and neurite outgrowth [21]. PC12 cells respond to NGF and when triggered, cease proliferation, extend neurites, and become electrically excitable [22].

There is, however, minimal information on the medicinal properties of *P. giganteus.* The aqueous and ethanolic extracts of *P. giganteus* have shown antioxidant, genoprotection (unpublished data) and liver protection properties [23]. To our knowledge, there are no reports on the nutritional composition of *P. giganteus* and its benefits on neurite outgrowth stimulation, if any. In the present study, aqueous and ethanolic extracts of *P. giganteus* fruiting bodies were investigated for their effects in neurite outgrowth of rat pheochromocytoma (PC12) cells. Prior to this, the cytotoxicity of the extracts was determined by using [3-[4,5-dimethythiazol-2-yl]-2,5-diphenyltetrazolium bromide] (MTT) assay. The hypothesis that MEK/ERK and PI3K/Akt are required for the neuronal differentiation and neurite outgrowth of PC12 cells was also tested using specific inhibitors.

## Methods

### Materials and chemicals

The fruiting bodies of *P. giganteus* were obtained from Nas Agro Farm, Sepang, Selangor, Malaysia. Rat pheochromocytoma (PC-12) cell line was purchased from American Type Culture Collection (ATCC; Rockville, MD, USA; Catalogue Number: CRL-1721.1TM). [3-[4,5-dimethythiazol-2-yl]-2,5-diphenyltetrazolium bromide] (MTT), phosphate buffered saline (PBS), dimethyl sulfoxide (DMSO), F-12 K medium (Kaighn’s Modification of Ham’s F-12 Medium), NGF-7 S from murine submaxillary gland, MEK inhibitor (U0126, PD98059), and PI3K inhibitor (LY294002) were obtained from Sigma Co. (St. Louis, MO, USA). Fetal bovine serum (FBS) and horse serum (HS) were purchased from PAA Laboratories (Cölbe, Germany).

### Cultivation condition of mushrooms

*Pleurotus giganteus* (KUM61102) was maintained on potato dextrose agar (PDA) at 4 - 10 °C and regularly subcultured. The substrate formulation for the cultivation of *P. giganteus* is similar to that for oyster mushroom cultivation, i.e. 89 - 94% (w/w) rubber wood sawdust, 5 - 10% (w/w) rice bran and 1% (w/w) calcium carbonate. Polypropylene bags are used for substrate bagging and the moisture content in the substrate was kept at 60% - 65%. The temperature for mycelia growth, spawn run, and fruiting body formation is 26 - 32 °C. Relative humidity of 70% and 80 - 90% during mycelia growth and fruiting; respectively, should be maintained. Direct illumination should be avoided as it has been reported to inhibit the fruiting body formation. A 20-day cycle after complete colonization of the artificial log is needed for each harvest and about four harvests (a total yield of 280 g) can be obtained from each bag of 900 g (Nas Agro Farm, personal communication).

### Cell culture

The PC12 cells (adherent variant, PC-12Adh) from ATCC were maintained in F-12 K medium (Sigma) supplemented with 2.5% (v/v) heat-inactivated fetal bovine serum (PAA) and 15% (v/v) horse serum (PAA) with final pH 6.8 - 7.2. All incubations were performed at 37 °C in a humidified environment of 5% CO_2_ and 95% air. The cells were maintained in the logarithmic phase of growth and were subcultured at 2–3 day intervals. For storage, the cells were frozen at −70 °C liquid nitrogen in complete medium supplemented with 5% (v/v) dimethyl sulfoxide (DMSO) (Sigma) as a cryoprotectant.

### Extraction of *P. giganteus* fruiting bodies

The fresh fruiting bodies were sliced, weighed and freeze-dried (Christ, Germany) for 1–2 days. The freeze-dried fruiting bodies were then ground using a blender. The resulting dried powder was weighed and kept in 4 - 8 °C. Aqueous extraction method was according to Eik et al. [8]. Briefly, the freeze dried powder was soaked in distilled water (1:20 ratio, w/v) and was left overnight at room temperature and 200 rpm in a shaker. The mixture was double boiled in water bath for 30 min and filtered (Whatman Grade 4) after cooling. The resulting aqueous extract was freeze-dried and kept at −40 °C prior to use. For ethanol extraction, the freeze dried powder was soaked in 95% ethanol at room temperature for three days and the process was repeated three times. The ethanol solvent was evaporated using a rotary evaporator (Eyela N-1000, USA) to give a brownish viscous extract.

### Nutritional composition of freeze dried fruiting bodies of *P. giganteus*

Fifty grams sample of *P. giganteus* fruiting bodies was sent to Consolidated Laboratory (M) Sdn. Bhd. for nutritional analysis.

### Cell viability and cytotoxicity assay

Cell viability and proliferation was determined by MTT assay [24]. Approximately 12,000 cells per well were seeded on a 96-well plate and incubated at 37 °C overnight in a humidified environment of 5% CO_2_ and 95% air. Fresh medium were then replaced and the cells were exposed to 0 to 1000 μg/ml of aqueous or ethanolic extract of *P. giganteus* for 48 hours. Subsequently, 20 μl of sterilized MTT (5 mg/ml) in phosphate buffered saline (PBS) buffer (pH 7.4) was spiked into each well and incubated at 37 °C for 4 hours. The supernatant was then carefully removed, and 200 μl of dimethyl sulfoxide (DMSO) was added into each well to dissolve the MTT formazan (blue colour) at the bottom of the wells. After 15 min, the absorbance at 540 nm with 690 nm as background absorbance was measured with an ELISA microplate reader (Sunrise, Tecan, Austria). The complete growth medium was the blank, and cells incubated in medium only without mushroom extracts were denoted as positive control.

### Neurite outgrowth stimulation activity

Neurite outgrowth stimulation assay was according to Eik et al. [8] with some modifications. The cells were seeded in a 6-well plate at an initial density of 5,000 cells per well in 2 ml complete growth medium with different concentrations of aqueous and ethanolic mushroom extracts. For freeze dried aqueous extract, a stock solution of 10 mg/ml was prepared freshly each time prior to assay. The stock solution was then diluted five times in sterile distilled water to final concentrations ranging from 5–100 μg/ml (w/v). For ethanolic extract, 10 mg/ml of stock solution in DMSO was prepared freshly. The solution was also diluted five times with sterile distilled water. In positive control experiments, cells were induced to differentiate by the addition of 50 ng/ml (w/v) NGF extracted from murine submaxillary gland (Sigma). Cells in complete growth medium only served as a negative control. All the cells were incubated for five days at 37 °C, 95% air and 5% CO_2_ to observe any neuronal differentiation activity.

### Quantification of neurite bearing cells

A cell was scored positive if it bears a thin neurite extension that was double or more the length of the cell body diameter [20]. Ten fields per well were randomly examined under an inverted microscope (Nikon Eclipse TS100). The cells were photographed using a Nikon DS-Fi1 camera and processed with a Nikon’s Imaging Software, NIS-Elements D. The percentage of neurite- bearing cells were quantified by scoring the number of neurite-bearing cells over the total number of viable cells in 10 microscopic fields with average of randomly chosen 200 to 300 cells per well.

### Treatment with specific inhibitors of signaling pathways

Stock solution (10 mM) of MEK inhibitor (U0126, PD98059) and PI3K inhibitor (LY294002) were prepared in DMSO and stored in −20 °C in the dark. Each inhibitor i.e. 10 μM for U0126 [25], 10–50 μM of LY294002 [26]; and 40 μM for PD98059 [27] was then prepared by diluting in medium just before use. PC12 cells were either incubated with or without the treatment of inhibitors for 1 hour. All the cells were then stimulated with 25 μg/ml of *P. giganteus* aqueous extract for three days prior to scoring neurite bearing cells.

### Statistical analysis

Results were expressed as the means ± standard deviation (SD). Data comparison between groups was performed using one-way analysis of variance (ANOVA). *P* < 0.05 was considered to be significant between groups by using Duncan's multiple range tests (DMRT).

## Results

### Nutritional composition of freeze dried fruiting bodies of *P. giganteus*

The nutritional components of *P. giganteus* fruiting bodies are shown in Table 1. *Pleurotus giganteus* contains 67.2 g/100 g of carbohydrate, 15.4 g/100 g of protein and 33.3 g/100 g of dietary fibre. It is rich in minerals like magnesium (67.64 mg/100 g) and potassium (1345.7 mg/100 g).

**Table 1:** **The breakdown of nutritional content of*Pleurotus giganteus*freeze-dried fruiting bodies**

**Test Parameter**	Result *	**Recommended daily allowance (RDA)**
Total Fat	3.7	65 g
·Saturated fat	0.97	-
·Monosaturated fat	1.97	-
·Polyunsaturated fat	0.77	-
·Trans fat	N.D (<0.01 g/100 g)	-
Energy in Kilo Calorie	364 kcal/100 g	-
Protein	15.4	50 g
Cholesterol	N.D (<0.001 mg/100 g)	300 mg
Carbohydrate	67.2	300 g
Dietary fibre	33.3	25 g
Sodium (as Na)	5.7	2400 mg
Calcium (as Ca)	5.78	1000 mg
Magnesium (as Mg)	67.64	0.4 g
Iron (as Fe)	1.85	18 mg
Zinc (as Zn)	2.68	15 mg
Phosphorus (as P)	526.45	700 mg
Potassium (as K)	1345.7	3500 mg
Copper (as Cu)	0.59	2.0 mg
Manganese (as Mn)	0.41	2.0 mg
Selenium (as Se)	N.D (<0.02 mg/kg)	70 μg

* g or mg/100 g of freeze-dried fruiting bodies; test method was according to AOAC (Association of Analytical Communities/Association of Official Agricultural Chemist); ND: Not detectable.

### The effects of aqueous and ethanolic extracts of *P. giganteus* on PC12 cell viability

**Figure 1:**
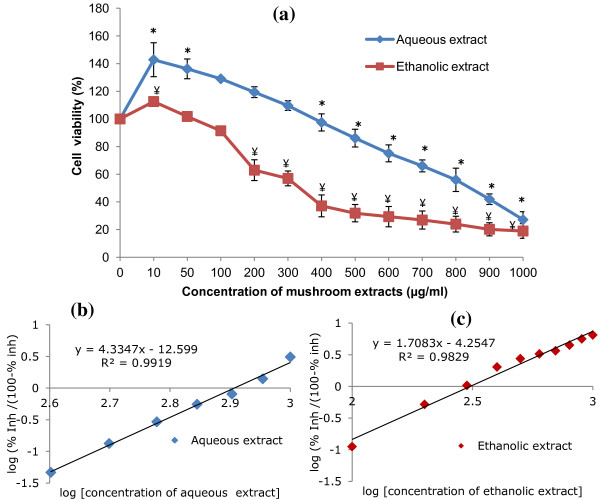
** The effects of aqueous and ethanolic extracts of*P. giganteus*on PC12 cell viability.** (**a**) Effect of aqueous extract and ethanolic extract on the cell proliferation of PC12 cells. The mean absorbance obtained using medium with cells only was designated 100%. Results shown represent the mean ± SD; *n* = 3. *^,¥^*p* < 0.05 for aqueous extract and ethanolic extract, respectively compared to the control 100%. (**b**) & (**c**): IC_50_ was obtained from the intercept on the x-axis (y = 0) of the regression line using the linear part of the percentage inhibition (% inh) curve (data not shown).

MTT assay was performed to determine the degree of cytotoxicity of *P. giganteus* extracts in PC12 cell. The cell viability and cell proliferation was denoted as 100% for the positive control i.e. cells in complete growth medium without mushroom extracts. It was shown that the growth of PC12 cell decreased with the increasing concentrations of the mushroom extracts. Figure 1a and the negative region of Figure 1b and 1c indicates that treatment with 10–200 μg/ml of aqueous extract and 10 μg/ml of ethanolic extract induced cell proliferation significantly (*p* < 0.05) as compared to control after a 48 h incubation. Upon challenge with a threshold dosage (500 μg/ml for aqueous extract and 200 μg/ml for ethanolic extract), the number of viable cells decreased significantly (*p* < 0.05) to 13.9% and 37.1%, respectively. At a concentration of 1000 μg/ml, the different extracts inhibited the cell proliferation to 75.65 ± 5.8% for aqueous extract, and 85.67 ± 5.3 for ethanolic extract. The IC_50_ which is the concentration at which 50% of cell growth inhibition occurs for aqueous extract and ethanolic extract were 806.39 ± 48 μg/ml and 309.46 ± 46 μg/ml, respectively. Hence, ethanolic extract is more toxic compared to aqueous extract, as the IC_50_ of ethanolic extract was 2.6-fold higher than that of aqueous extract.

### The effects of aqueous and ethanolic extracts of *P. giganteus* on neurite outgrowth of PC12 cells

**Figure 2:**
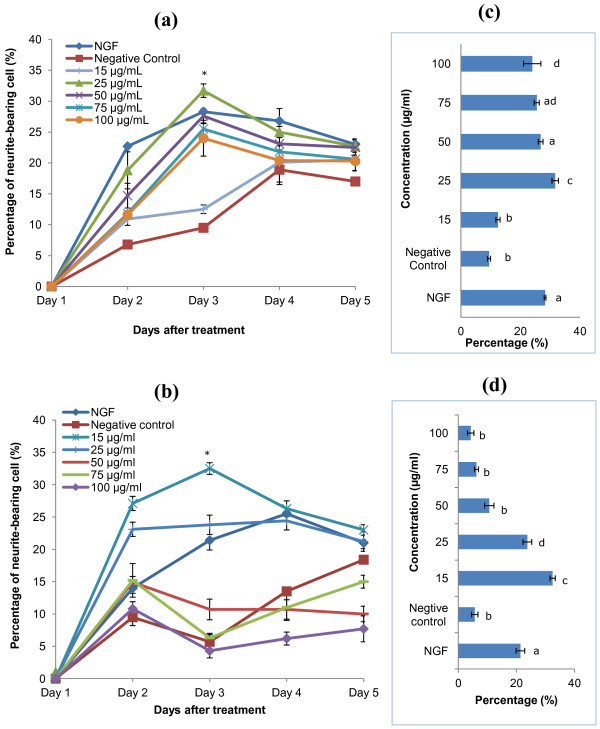
** The effects of aqueous and ethanolic extracts of*P. giganteus*on neurite outgrowth of PC12 cells.** (**a**) & (**b**) Time- and dose-dependent neurite outgrowth induced by aqueous extract and ethanolic extract, respectively. * *p* < 0.05 compared with positive control NGF and negative control. (**c**) & (**d**) Percentage of neurite-bearing cells on day 3 for aqueous extract and ethanolic extract, respectively. Results shown represent the mean ± SD; *n* = 5. Means not sharing a common letter were significantly different at *p* < 0.05.

All concentrations of mushroom extracts tested were non-cytotoxic to the cells, as determined by MTT assay. Aqueous extract of *P. giganteus* induced neurite outgrowth of PC12 cells in both a time- and dose-dependent manner (Figure 2a). On the second day, the percentage of neurite-bearing cells increased significantly (*p* < 0.05) to 18.8% after treatment with 25 μg/ml of aqueous extract when compared to time-matched negative control (9.5%). After stimulation with aqueous extract, the percentage of neurite-bearing cells significantly increased (*p* < 0.05) until the effect reached a plateau after day 3. Therefore, day 3 was selected for further studies as the neurite scoring for all concentrations were the highest. Similarly, ethanolic extract induced neurite outgrowth of PC12 cells in a time- and dose-dependent manner and the number of neurite-bearing cells remained constant after day 3, as shown in Figure 2(b).

**Figure 3:**
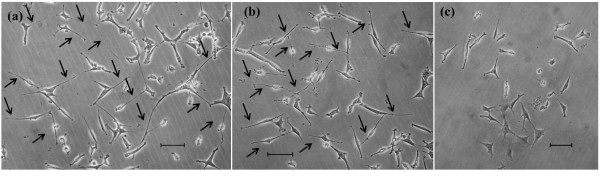
** Phase-contrast photographs of PC12 neurites at day 3.** (**a**) Treatment with 50 ng/ml NGF (**b**) Treatment with 25 μg/ml of aqueous extract, and (**c**) Negative control, treatment of nither of (**a**) and (**b**). Scale bar = 20 μm. Arrows indicate neurite extentions.

Figure 2c and 2d give the percentage of neurite-bearing cells for aqueous extract and ethanolic extract, respectively, on day 3. As shown in Figure 2c, aqueous extract at 25 μg/ml had a significant (*p* < 0.05) effect (31.7 ± 1.1%) in stimulating neuronal differentiation compared to NGF (28.3 ± 0.4%). On day 3, 15 μg/ml of ethanolic extract induced 33.3 ± 0.9% of neurite-bearing cells (Figure 2d). There was no significant difference (*p* > 0.05) in the percentage of neurite-bearing cells at 25 μg/ml of aqueous extract and 15 μg/ml of ethanolic extract. However, both the extracts performed better than NGF (*p* < 0.05). It was obvious for ethanolic extract, that 50 μg/ml, 75 μg/ml and 100 μg/ml did not significantly (*p* > 0.05) trigger neuronal differentiation and neurite outgrowth of PC12 as compared to aqueous extract for the same concentrations. Figure 3 shows the morphology of PC12 cells with neurites at day-3 of treatment with 50 ng/ml NGF (a), 25 μg/ml of aqueous extract (b), and neither of them (c).

### The mechanism of neurite outgrowth stimulation by the extracts of *P. giganteus*

**Figure 4:**
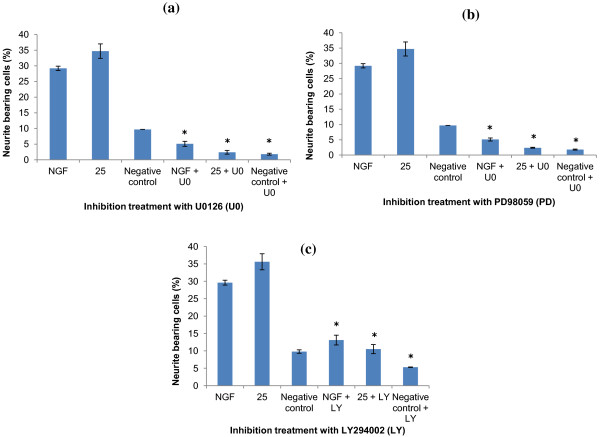
** Comparison of the percentage of neurite-bearing cells with different specific inhibitors treatment.** (**a**) U0126 (10 μM), (**b**) PD98059 (40 μM), and (**c**) LY294002 (30 μM). NGF, 25 μg/ml of aqueous extract (denoted simply by 25), and negative control were the control groups i.e. without the treatment with inhibitors. Results shown represent the mean ± SD; *n* = 3. * Significant difference at *p* < 0.05 versus control group.

It was shown that neurite outgrowth induced by NGF and aqueous extract of *P. giganteus* was markedly inhibited (*p* < 0.05) by MEK inhibitors U0126 and PD98059 (Figure 4a and 4b). In fact, in PC12 cell treated with aqueous extract combined with either 10 μM of U0126 or 40 μM of PD98059, the decrease in the number of neuritic processes was significant (*p* < 0.05). On the contrary, an inhibitor of PI3K/Akt pathway, LY294002, did not inhibit aqueous extract- and NGF-induced neurite outgrowth at the concentration of 10 μM and 20 μM (*p* > 0.05). LY294002 at the concentration of 30 μM started to cause inhibition effects on PC12 in a concentration-dependent manner. At 30 μM of LY294002, the number of elongated PC12 cells with neurites doubled the cell diameter decreased significantly, by 49.6% and 63.5%, for NGF- and aqueous extract-treated cells; respectively (Figure 4c). At 50 μM, all the cells pre-treated with the inhibitor showed no difference (*p* > 0.05) to the negative controls, with differentiated cells bearing neurites ranging only from 3.2 – 5.3%. From this result, we proposed that aqueous extract induced neurite outgrowth on PC12 cells via the activation of ERK1/2 cascade and PI3K/AKt pathways.

## Discussion

There is a vast amount of nutritional studies of wild and cultivated mushrooms across the world. However, relatively little data exist in the literature on the nutrient content of *Pleurotus giganteus*. Herein, it was intended to compare only the highly appreciated and most cultivated culinary-medicinal mushrooms, for example the *Pleurotus* genus and *Agaricus* genus. Generally, mushrooms have high (19 – 35%) protein contents [28]. In Brazil, it was reported that the fruiting bodies of *Pleurotus ostreatus* and *Pleurotus sajor-caju* presented protein content ranging from 13.1% to 18.4%, depending on the substrates used [29]. The present study showed that the protein level of *P. giganteus* is 5.3-time lower than that of *Agaricus bispo*r*us* (white button mushroom) with reference to a study from Portugal [30]. On the other hand, the carbohydrate content in *P. giganteus* is 4-, 6-, 7.2-, 7.5-, 8-, 11-time higher than that of *Lentinula edodes*, shiitake (17.12 g/100 g), *Flammulina velutipes*, golden needle mushroom (10.57 g/100 g), *Pleurotus ostreatus,* oyster mushroom (9.30 g/100 g), *Pleurotus eryngii*, king oyster mushroom (8.95 g/100 g), *Agaricus bisporus* white button mushroom (8.25 g/100 g) (30), and *Agaricus bisporus* brown mushroom (5.98 g/100 g) [31]. This suggested that carbohydrates (glucose, mannitol, trehalose, oligosaccharide groups, and reserved polysaccharide like glycogen) account for the prevailing component of *P. giganteus* fruiting body. Reports related to the nutritional evaluation of *Pleurotus* genus carried out by other researchers from different regions (Japan, India, Bangladesh, Turkey, Finland, and Italy) can be retrieved from [32-37], respectively. Nevertheless, the differences between the nutrient values may be attributed to the type of mushroom, strain of mushroom, environmental factors, and composition of growth media [37].

MTT assay is by far the most convenient colorimetric assay based on the metabolic activity of a viable cell [24,38]. Basically, only viable cell has the mitochondrial dehydrogenase system that can cleave the yellow MTT tetrazolium salt and yield MTT formazan which is blue in colour. Thus, the optical density of the amount of solubilised MTT formazan is quantitatively correlated to the percentage of cell viability. The present study showed that cytotoxic effect of *P. giganteus* aqueous and ethanolic extracts towards PC12 cells were concentration dependant. This is consistent with the finding by Cheung et al. [39] whereby viability of PC12 cells was dose-dependently decreased by increasing *Ganoderma lucidum* extracts.

On-going studies show that the aqueous extract of *P. giganteus* contains bioactive secondary metabolites like sterols and triterpenes (unpublished data). These compounds are reported to have neutrophic NGF-like properties and caused neurite outgrowth activity in PC12 cells [40]. We have shown for the first time that *P. giganteus* extract can stimulate neurite outgrowth by using PC12 cell line model. It was shown that 25 μg/ml of aqueous extract and 15 μg/ml of ethanolic extract induced the highest percentage of neurite outgrowth in PC12 cells at day 3. The number of neurite bearing cells was significantly higher than that of NGF. The mushroom extracts may contain bioactive compounds either mimic NGF or trigger the production of NGF, hence resulting in neurite outgrowth. Further, the potassium level in *P. giganteus* was 1345.7 mg/100 g and according to Kalac [41], potassium level in fruiting bodies is between 20- and 40-fold higher than in the substrates used for mushroom cultivation. In the study by Cohen-Cory et al. [42], the cell number of Purkinje cells, the major efferent neurons of the brain cerebellum increased by 40% when treated with potassium. Besides, potassium alone or potassium coupled with NGF markedly increased the cell survival, cell differentiation and neurite outgrowth. In this study, the potassium present in *P. giganteus* extracts may be involved in the regulation of the morphological differentiation of PC12 cells by acting as a depolarising agent.

The present study extends recent findings that some mushroom extracts can have neuritogenesis effects. Prior studies by our group have shown that 0.2% (v/v) aqueous extract of freeze dried fruiting bodies from *Hericium erinaceus* caused maximal stimulation of neurite outgrowth (17.3% of neurite bearing cells and 88.2% increase compared to control) in NG108-15 cell line after 24 hours of incubation [5]. Besides, freeze drying was found to be the best approach to preserve the bioactive compounds in mushroom as compared to oven-dried method [43]. It had been reported that PC12 cells responded well to water extract of sclerotium of *Lignosus rhinocerus* (Cooke) Ryvarden [8]. It was found that synergistic effect, i.e. 42.12% of neurite bearing PC12 cells was elicited when the cells were treated with 20 μg/ml of water extract combined with 30 ng/ml of NGF. Some other medicinal mushrooms that induced neurite outgrowth included *Grifola frondosa* (Maitake) [10], *Tricholoma* sp [44], *Termitomyces albuminosus*[45,46]*, Dictyophora indusiata*[47]*, Tremella fuciformis*[48], and *Ganoderma lucidium* (Lingzhi) [39].

The involvement of the MAPK/ERKs signaling pathway in neuronal differentiation by mushroom extracts has been reported. Neuroprotective and neuritogenesis effect of *Ganoderma lucidium* extracts on PC12 was stipulated to be mediated via the MAPK/ERK signalling pathway [39]. Besides, lysophosphatidylethanolamine from *Grifola frondosa* induced activation of ERK1/2 of PC12 cells thus stimulated neurite outgrowth and inhibited serum withdrawal-induced apoptosis [10]. Neurotrophins like NGF are mostly mediated by the Trk family of receptor tyrosine kinase, TrKA. However, discrepancy did occur in the case of *Ganoderma lucidium* extracts, whereby there was no direct involvement of TrkA [39]. Similarly, *α*-Phenyl-*N-tert*-butylnitron was also found to induce neurite outgrowth in PC12 independent of TrkA [49]. It is thus predicted, based on the ability of *P. giganteus* extract to stimulate neurite outgrowth of PC12 without NGF, that activation of TrKA receptor tyrosine kinase may not be necessary. According to Sweatt [50], the mitogen-activated protein kinase (MAP kinase, MAPK) cascade is a superfamily of signalling cascade and is a vital regulator of cell division and differentiation. Recently, MAPK was specified as the extracellular signal-regulated kinase (ERK) comprising ERK 1 and 2, or as ERK1/2. It has been demonstrated that ERK-cascade was necessary and sufficient enough for NGF-induced neuronal differentiation of PC12 cells. In the present study, upon inhibition by MEK-selective inhibitor U0126 and PD98059, the percentage of neurite outgrowth decreased significantly. This suggested that ERK1/2 phosphorylation was affected and this indirectly implied that activation of ERK1/2 is necessary for *P. giganteus*-mediated neuritogenesis. Inhibition of PI3K/Akt signalling by LY294002 also negatively affected neurite outgrowth of PC12. This finding suggested that neurite outgrowth potentiated by *P. giganteus* in PC12 cells is also regulated by PI3K/Akt signaling pathway. However, it was noted that PI3K/Akt inhibitor did not markedly affect the activities of ERK [51], therefore neurite extension of PC12 still could be observed at lower concentrations of LY294002. According to Naidu et al. [52], phospho-Akt and phospho-MAPK were expressed during neurodevelopment and nerve regeneration following sciatic nerve crush on rats. Collectively, these results demonstrated that *P. giganteus*-induced neurite extension is regulated at least by part between MEK/ERK and PI3K/Akt pathways. For the future work, confirmation by immunoblot analysis to detect the phosphorylation of TrKA, ERK, and Akt, is necessary.

## Conclusions

To our knowledge, this is the first evidence on the effects of *Pleurotus giganteus* aqueous and ethanol extracts on neuronal differentiation and neurite outgrowth. The high potassium level in the fruiting bodies and the presence of bioactive compounds (mainly triterpenoids) could be responsible for the neuroactivity. Work is in progress to determine and identity the bioactive compound/s responsible for the activity. Our results suggested that neurite outgrowth stimulated by *P. giganteus* is mediated via the “cross-talk” between MEK/ERKs and PI3K/Akt pathways. However, further immunoblot analysis is required.

## Competing interest

The authors declare that they have no competing interests.

## Authors’ contributions

CWP carried out the experiment, drafted the manuscript, and engaged in data acquisition and data interpretation. WLW carried out ethanol extraction, and preparation of samples for nutritional analysis. PD participated in the acquisition of funding and editing for manuscript. MN involved in the design of the study and manuscript editing. VS provided the grant, involved in coordinating and monitoring of research; and manuscript editing. All authors read and approved the final manuscript.

## Pre-publication history

The pre-publication history for this paper can be accessed here:

http://www.biomedcentral.com/1472-6882/12/102/prepub
